# Case report: Heterozygous mutation in HTRA1 causing typical cerebral autosomal recessive arteriopathy with subcortical infarcts and leukoencephalopathy

**DOI:** 10.3389/fgene.2023.1235650

**Published:** 2023-09-18

**Authors:** Yu-Ming Li, Wei Jia, Tao Xin, Yu-Qing Fang

**Affiliations:** ^1^ Department of Neurosurgery, The First Affiliated Hospital of Shandong First Medical University & Shandong Provincial Qianfoshan Hospital, Jinan, Shandong, China; ^2^ Department of Gastroenterology, The First Affiliated Hospital of Shandong First Medical University & Shandong Provincial Qianfoshan Hospital, Jinan, Shandong, China; ^3^ Medical Science and Technology Innovation Center, Shandong First Medical University and Shandong Academy of Medical Sciences, Jinan, China; ^4^ Department of Neurosurgery, Jiangxi Provincial People’s Hospital, Nanchang, Jiangxi, China; ^5^ Post-Doctoral Scientific Research Station, Shandong University of Traditional Chinese Medicine, Jinan, Shandong, China; ^6^ Department of Neurology, The First Affiliated Hospital of Shandong First Medical University & Shandong Provincial Qianfoshan Hospital, Jinan, Shandong, China

**Keywords:** cerebral autosomal recessive arteriopathy with subcortical infarcts and leukoencephalopathy, high-temperature requirement serine peptidase A1, heterozygous, mutation, stroke

## Abstract

**Background:** Cerebral autosomal recessive arteriopathy with subcortical infarcts and leukoencephalopathy (CARASIL) is an autosomal recessive disorder characterized by baldness, recurrent ischemic stroke, lumbago, headache, and dementia which is closely related to homozygous mutations of the high-temperature requirement serine peptidase A1 (HTRA1) gene. Heterozygous mutations of HTRA1 are usually considered to be non-pathogenic. Although it has been revealed that only a few patients with heterozygous mutations could present some manifestations, their clinical symptoms were atypical, milder, and always with a lower frequency of extra-neurological features. Here, a rare patient with heterozygous mutation of HTRA1 who had all typical features of CARASIL as well as severe clinical symptoms and rapid progression was initially reported in our study.

**Case presentation:** A 43-year-old female patient presented with a gradual onset of headache and cognitive decline. As time progressed, her headache intensified and symptoms of dementia began to manifest gradually. During her early years, she had thinning hair and subsequently experienced two occurrences of ischemic strokes in her thirties. Furthermore, she also had a history of lumbago and urinary retention before visiting our hospital. The patient’s magnetic resonance imaging revealed the presence of widespread white matter lesions, infarctions, and microbleeds, in addition to lumbar disc herniation and degenerative lesions. The observed clinical characteristics had a strong correlation with CARASIL, and the patient was diagnosed with a heterozygous missense mutation of 905G>A (Arg302Gln) in the HTRA1 gene. The patient has been under continuous follow-up for a duration exceeding 3 years subsequent to her release from the hospital. She underwent cystostomy, and symptoms of bulbar paralysis developed in a progressive way. Currently, there has been a notable decrease in motor function and activities of daily living, resulting in the individual being confined to bed for a duration exceeding 1 year.

**Conclusion:** This case suggests that patients carrying a heterozygous mutation in G905A may also have typical clinical features of CARASIL, which allows us to have a more comprehensive understanding of CARASIL.

## Introduction

Cerebral autosomal recessive arteriopathy with subcortical infarcts and leukoencephalopathy (CARASIL) is a rare inherited cerebral small vessel disease (CSVD) which is associated with mutations of the high-temperature requirement serine peptidase A1 (HTRA1) gene (Devaraddi et al., 2018). It is commonly characterized by baldness, recurrent ischemic stroke, cognitive impairment, spinal deformity, lumbago, and headache (Tikka et al., 2014). Cases of this disease have predominantly been documented in Japan, with a limited number of individuals identified in other nations in recent years (Nozaki et al., 2016). CARASIL is an autosomal recessive disease caused by homozygous mutations of HTRA1, and heterozygous mutations are often considered to be non-pathogenic. However, there is some evidence suggesting that heterozygous mutations in the HTRA1 gene may be related with CSVD (Wu et al., 2018).

Studies have revealed that distinct mutation locations can lead to a reduction or complete loss of serine protease activity in HTRA1 through diverse pathways (Yao et al., 2022). *In vitro*, the fluorogenic assay revealed that a mixture of mutant and wild-type HTRA1 in Freestyle 293 cells showed decreased protease activity (Sun et al., 2022). It is widely recognized that the majority of individuals who carry heterozygous mutations of HTRA1 do not exhibit any clinical symptoms. However, it remains unclear why some clinical symptoms could appear in a very few carriers. These symptomatic individuals with heterozygous mutations presented low protease activity, with mild clinical manifestation, and only a portion of CARASIL symptoms with slow progression (Fukutake, 2011). In contrast to previously documented cases, we initially report an exceptional individual with a heterozygous mutation of HTRA1 who exhibited nearly all the classic features of CARASIL along with severe clinical manifestations and rapid progression.

## Case report

### History and presentation

**FIGURE 1:**
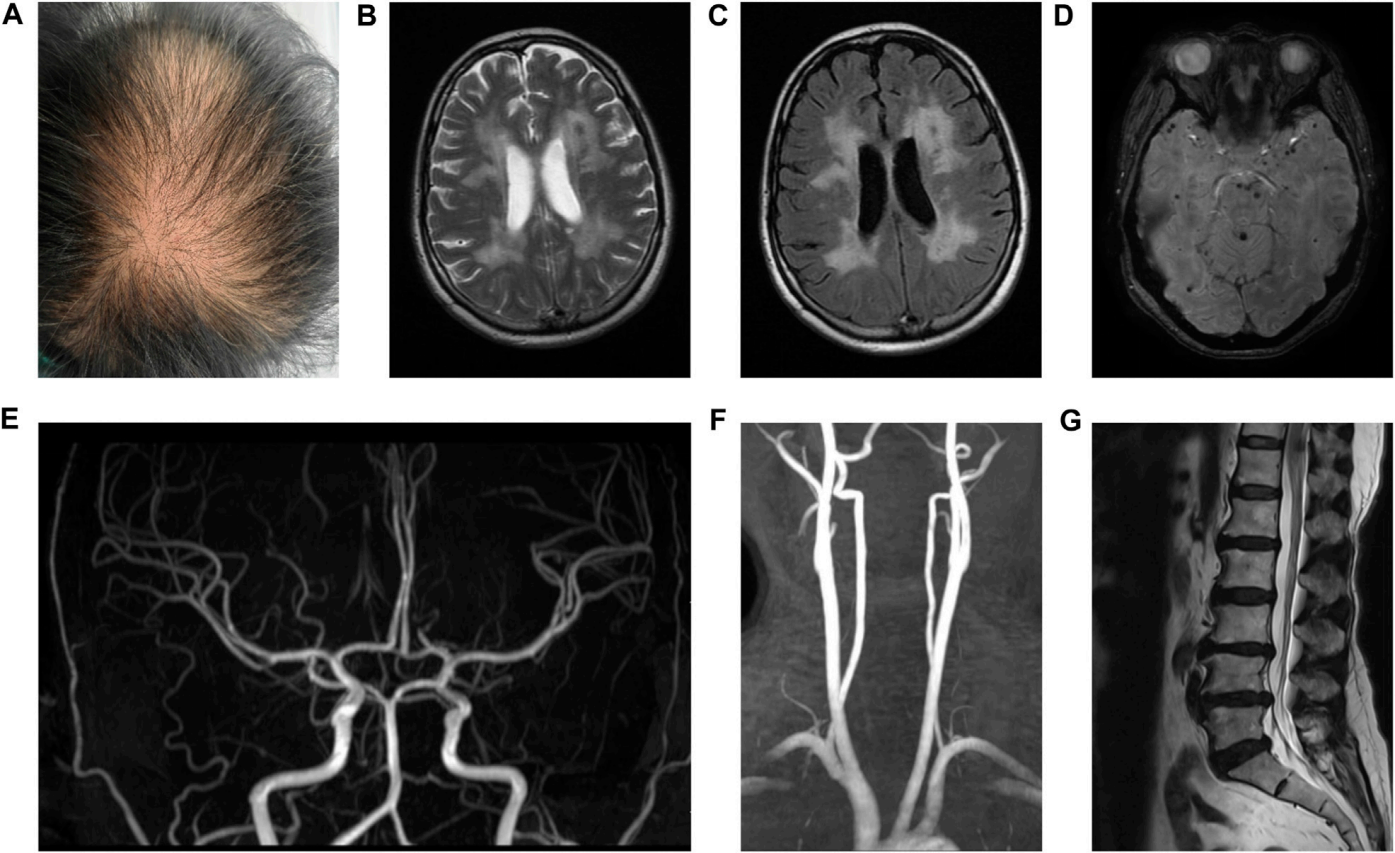
Hair and MRI of the patient. Hair of the patient **(A)**. T2W and flair images revealed diffuse white matter lesions and ischemic infarctions **(B and C)**. SWI showed microbleeds in the brain parenchyma **(D)**. MRA showed normal intracranial arteries **(E)** and carotid arteries **(F)**. T2W image showed lumbar disc herniation and degenerative lesions **(G)**.

This case involved a 43-year-old female patient. She was an office worker and visited our hospital in 2020 with the chief complaint of 3 years of headache and more than 1 year of cognitive disorder. She has been afflicted by migraine without aura since 2017. Initially, the migraine was paroxysmal in nature, with each episode lasting for a duration of a few minutes to half an hour. Nevertheless, it gradually worsened and exhibited a higher frequency together with prolonged durations. Suffering from headache for more than 1 year, she developed memory decline and slow cognitive response. In the beginning, she experienced difficulties in recalling information, followed by cognitive impairments such as emotional indifference, abnormal communication, and a tendency toward reticence. In retrospect, there were no infectious illnesses prior to her onset. She was subjected to ischemic strokes twice in her thirties, causing slow movements and weakness of limbs. Moreover, lumbar spondylosis and lumbago have affected her for 5 years, and urinary retention hit 6 months ago. Low back pain sometimes manifested as an acute episode, characterized by an unpredictable start and variable length spanning from a few minutes to several days. In severe cases, painkillers were needed. She has exhibited thinning hair since childhood. No history of chronic diseases, such as hypertension, diabetes, and cardiovascular disorders, was found. Her mother suffered from moderate cerebral infarction and lumbar disc herniation, whereas her father tragically died suddenly as a result of an accident although maintaining good health throughout his life. The patient was an only child and raised two healthy daughters. After admission, she went through physical examinations which revealed normal blood pressure and thinning hair particularly on the crown of her head (Figure 1A). There was a decline observed in the capacities of response, calculation, and memory retention. Her Montreal Cognitive Assessment (MoCA) score was recorded as 16 and Mini-Mental State Examination (MMSE) score was noted as 19. Her movements were slow, presenting staggering walk with a spastic gait. In addition, the strength of her right arm and two legs was weakened. Babinski signs of both sides were positive. The sensory examination and assessment of tendon reflexes were predominantly normal. No abnormal findings were detected in the blood tests, which encompassed immunological indicators, blood routine, blood sugar, blood lipids, antiphospholipid antibodies, platelet function, and other relevant parameters. The results of the cerebral fluid examination indicated normal findings, with no presence of oligoclonal bands. Diffuse white matter lesions, infarctions, and microbleeds were observed in the magnetic resonance imaging (MRI) of her brain (Figures 1B–D). Meanwhile, magnetic resonance arterial imaging exhibited fundamental normalcy (Figures 1E, F). Lumbar spine MRI revealed the presence of lumbar disc herniation and degenerative lesions (Figure 1G). Her MRI findings should be differentiated from demyelinating diseases. Distinguished from the aforementioned disease, the presence of microbleeds was seen on susceptibility-weighted imaging. In addition, there were no detected instances of previous infection, relapsing-remitting courses, or immunological abnormalities in the cerebrospinal fluid and serum, which effectively eliminated the possibility of demyelinating disorders. Ultrasound indicated that her cardiac structure was normal. The patient presented with characteristic clinical symptoms of ischemic stroke, headache, lumbar spondylosis, and baldness, which were observed at a rather early age. Furthermore, the patient exhibited cognitive impairment and urine retention, both of which could also be discovered in individuals diagnosed with CARASIL. Based on an analysis of the patient’s medical history, symptoms, physical signs, and MRI, a diagnosis of CARASIL was determined in accordance with the diagnostic criteria established by Fukutake (2011). CARASIL is a hereditary disorder for which there is currently no established or targeted therapeutic intervention. The majority of patients do not have risk factors associated with cerebrovascular illness such as hypertension and diabetes, and other related conditions. Consequently, the patient received a comprehensive symptomatic treatment regimen consisting of antiplatelet medications, statins, and donepezil. Her conditions remained stable during hospitalization.

### Sequencing

**FIGURE 2:**
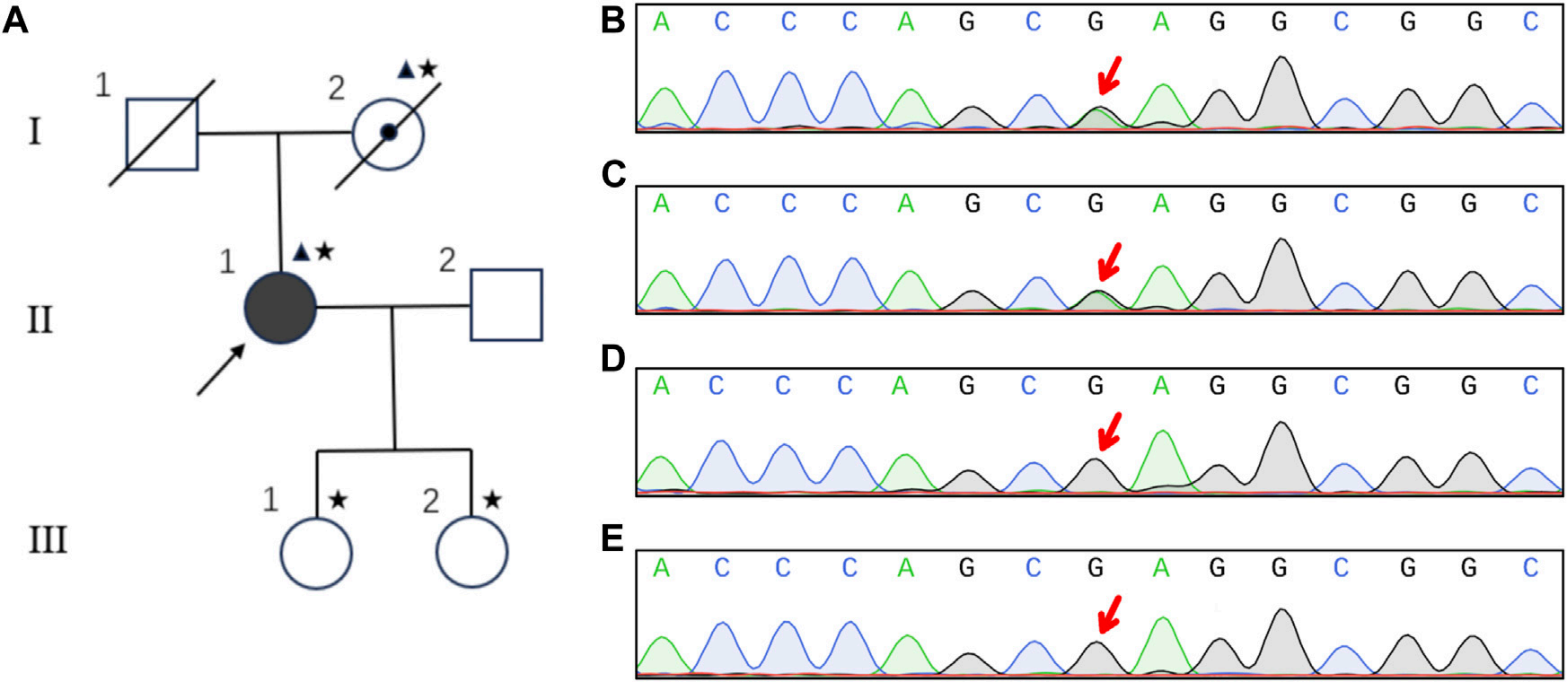
Pedigree and sequencing results of the family. Pedigree of the family **(A)**. Square, male; circle, female; diagonal black line, deceased individual; center dot symbol, individual with atypical and mild clinical symptoms; full black-filled symbol, individual affected by typical and severe clinical symptoms; empty symbol, clinically healthy relative; triangle, individuals with heterozygous mutation; asterisk, members undergoing genetic testing; arrow, proband. The heterozygous mutation of G905A in HTRA1 was detected in the patient **(B)** and her mother **(C)**. Her two daughters were normal **(D and E)**.

The pedigree is shown in Figure 2A. Given the diagnosis of CARASIL was established, whole exome sequencing as well as CNV-seq was employed for the diagnosis of this patient. A heterozygous missense mutation c.905G>A (p.Arg302Gln) was detected (Figure 2B), and the variant was likely pathogenic according to the ACMG classification (Mahale et al., 2021). No Notch 3 mutation was observed. Her mother carried the same mutation (Figure 2C) but her two daughters did not (Figures 2D, E).

### Follow-up

Following the patient’s release, a comprehensive follow-up has been conducted for a duration exceeding 3 years. Her conditions aggravated as time went by. She subsequently experienced cerebral infarction many times, resulting in a further deterioration of her physical activity and verbal function, and signs of dementia were increasingly conspicuous. Nine months after leaving the hospital, she underwent cystostomy, after which symptoms of bulbar paralysis such as coughing and swallowing difficulties were developed in late 2021. As a result, she became reliant on nasal feeding. Currently, there has been a notable deterioration in her motor function and activities of daily living, leading to a state of being confined to bed for a duration exceeding 1 year. Her mother passed away at the age of 64 years in 2021 due to intestinal blockage. Her two daughters, on the other hand, had no clinical signs and were considered to be in a normal state.

**FIGURE 3:**
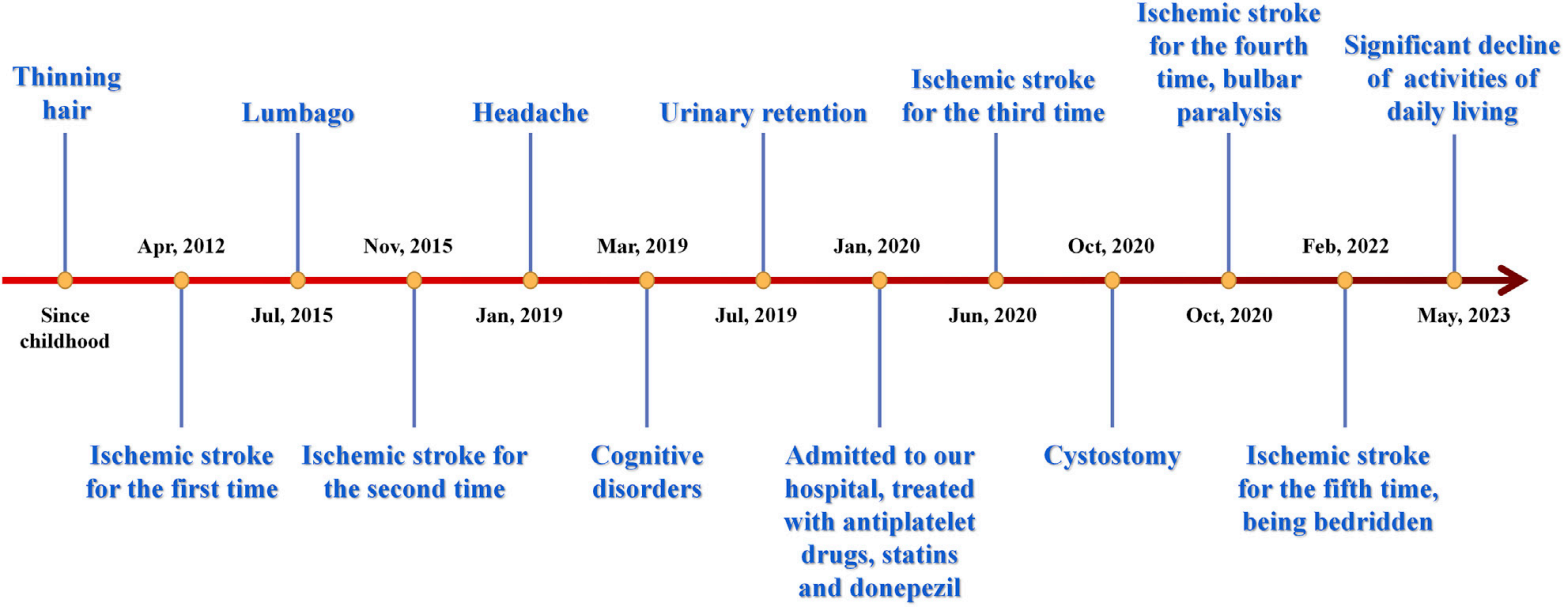
Time axis of patient’s clinical symptoms and relevant therapies. IS, ischemic stroke.

Figure 3 displays a temporal axis depicting the patient’s clinical symptoms and the corresponding therapies administered to her. Overall, her conditions progressed rapidly, and the medication’s efficacy was found to be limited, resulting in significant distress for both the patient and her family members. There was concern over the potential exacerbation of the illness.

## Discussion

CARASIL is an autosomal recessive inherited disease initially documented by a Japanese scholar in 1965 and officially named by Fukutake and Hirayama (1995). Homozygous mutations in the HTRA1 gene are responsible for the onset of the disease, whereas heterozygous mutations are usually considered to be non-pathogenic. The summary of variants identified in patients with HTRA1-related CARASIL is shown in Table 1. CARASIL is characterized by an earlier onset and the presence of distinctive non-neurological manifestations. Stroke often occurs at an early stage and exhibits a rapid progression, typically lacking the presence of major risk factors associated with cerebrovascular diseases such as hypertension and diabetes (Diwan et al., 2012). A majority of patients encounter recurring ischemic strokes, with lacunar infarction being the most common subtype. Recurrent episodes of infarction invariably result in the gradual deterioration of cerebral functions and finally culminate in dementia between the age of 30 and 45 years. Approximately 80% of CARASIL patients suffer from low back pain because of disk herniation and spondylosis deformans (Al et al., 2022). The majority of heterozygous HTRA1 carriers were asymptomatic, and only a few patients were symptomatic (Hara et al., 2009). Research has revealed that individuals with symptomatic HTRA1 heterozygous variations were likely to experience the beginning of symptoms at a later age compared to those with CARASIL. Moreover, these individuals might exhibit milder clinical manifestations as well as a delayed development of stroke and dementia. Meanwhile, it was consistently observed that they had a reduced incidence of extra-neurological symptoms such as baldness and spinal disorders (Tikka et al., 2014). However, the patient in our study was quite different from the individuals published previously, as she presented very severe clinical symptoms, rapid progression, and was highly consistent with the typical characteristics of CARASIL.

**TABLE 1:** Summary of variants identified in patients with HTRA1-related CARASIL.

No	Nucleotide change	Amino acid change	Mutation type	Patients	Families	References
1	889G>A	V297M	Missense	5	2	Hara et al. (2009)
2	754G>A	A252T	Missense	2	1	Hara et al. (2009)
3	821G>A	R274Q	Missense	3	2	Nishimoto et al. (2011)
4	1091T>C	L364P	Missense	2	1	Wang et al. (2012)
5	126delG/961G>A	Q42fs/A321T	Compounds heterozygous	1	1	Bianchi et al. (2014)
6	161_162insAG	G56fs	Frameshift	1	1	Cai et al. (2015)
7	517A>G	A173T	Missense	1	1	Khaleeli et al. (2015)
8	1005+1G>T	NA	Splice site	1	1	Roeben et al. (2016)
9	616G>A	G206R	Missense	1	1	Ibrahimi et al. (2017)
10	739delG	E247Rfs	Frameshift	1	1	Preethish-Kumar et al. (2017)
11	830_831delAG	E227Vfs	Frameshift	1	1	Preethish-Kumar et al. (2017)
12	502A>G	K168X	Nonsense	1	1	Preethish-Kumar et al. (2017)
13	958G>A/1021G>A	D320N/G341R	Compounds heterozygous	1	1	Xie and Zhang (2018)
14	983C>A	S328X	Nonsense	1	1	Gündüz et al. (2019)
15	805insG	S270Lfs*69	Frameshift	3	1	Ziaei et al. (2019)
16	847G>T	G283X	Missense	1	1	Yu et al. (2020)
17	472+2 T>C	NA	Splice site	1	1	Sun et al. (2022)
18	1156C>T	R386X	Nonsense	2	1	Shirah et al. (2023)

CARASIL, cerebral autosomal recessive arteriopathy with subcortical infarcts and leukoencephalopathy; NA, not available/not applicable.

The pathogenesis of CARASIL is related to homozygous or compound heterozygous mutations, which ultimately lead to the functional impairment of HTRA1 (Kaur et al., 2021). Hara et al. (2009) demonstrated that homozygous mutation could result in protein products with clearly lowered protease activity, and patients with homozygous mutation presented typical CARASIL symptoms. HTRA1 belongs to the family of serine protein induced by heat shock and is highly conserved in evolution. The HTRA1 protein is composed of several distinct domains, including a signal peptide domain, an insulin-like growth factor binding protein domain, a Kazal serine protease inhibitor domain, a trypsin-like serine protein domain, and a PDZ domain. HTRA1 is a serine protease that forms homotrimers, with each HTRA1 subunit activating the adjacent HTRA1 *via* the sensor domain of loop 3 (L3, amino acid positions 301–314) and the activation domain of loop D (Zurawa-Janicka et al., 2017). Heterozygous R302Q HTRA1, meanwhile, forms trimers, and the mutation is located in L3 domain, which is important for trimer-associated HTRA1 activation (Nozaki et al., 2016). In the L3 domain, R302 is the only portion observed in the symptomatic carrier. R302 is essential for intermonomer communication and substrate binding (Cabrera et al., 2017). Hence, the occurrence of a mutation in the L3 domain that disrupts the process of signal transduction between the monomers may lead to the inhibition of the wild-type protease activity. The wild-type protease activity was interfered by the trimer-dependent activation cascade, which resulted in less than 50% of protease activity in symptomatic carriers (Nozaki et al., 2016). So the heterozygous variant has a dominant negative effect on the protease activity of wild-type HTRA1 involved in a stable trimer (Fasano et al., 2020; Yao et al., 2022). In addition, the mutated HTRA1 gene leads to reduced serine protease activity, which is unable to inhibit the TGF-β signaling pathway; the overexpression of this pathway promotes the deposition of extracellular matrix (ECM). The excessive accumulation of ECM further leads to the proliferation of vascular intima, division of the inner elastic layer, and degeneration of vascular smooth muscle, therefore contributing to the development of cerebral arteriolar stenosis in CARASIL. Then the clinical neurological symptoms and the corresponding extensive white matter lesions in imaging could be found (Yokobori and Nishiyama, 2017).

An investigation indicated that the R302Q variant *in vivo* could result in a mutant protein with 21%–50% of normal protease activity and was associated with increased TGF-β signaling in the small cerebral arteries (Hara et al., 2009). The aforementioned mutation was also identified in two Japanese individuals who were not related to each other and exhibited CSVD presenting with subcortical lacunar infarcts with a later age at onset (49–59 years) and no extra-neurological features (Ohta et al., 2020). Every instance of a heterozygous HTRA1 missense mutation has a distinct and unique pattern of HTRA1 expression. The patient reported in our study was quite different from previously published research participants, who presented with very severe clinical manifestations of CARASIL. One potential reason is that the patient’s heterozygous mutation may exert a significant inhibitory effect on protease activity. Moreover, variations in penetrance can potentially serve as an alternative explanation. For individuals carrying genetic mutations, some are completely normal, some only exhibit very mild or atypical features, and others can develop clinical diseases with typical symptoms. These suggest that incomplete penetrance is frequent and the effects of epigenetic modifications should be considered.

In conclusion, we reported an individual with heterozygous mutation in HTRA1 who had typical characteristics and severe symptoms of CARASIL with rapid progression. This is an extremely rare and particular case since it contributes to the advancement of our entire comprehension of CARASIL. Extensive genetic screenings and in-depth experiments of heterozygous HTRA1 variants are needed for further studies.

## Data Availability

The datasets for this article are not publicly available due to concerns regarding participant/patient anonymity. Requests to access the datasets should be directed to the corresponding authors.
